# A Rare Case of Vaping-Induced Spontaneous Pneumomediastinum

**DOI:** 10.7759/cureus.17166

**Published:** 2021-08-13

**Authors:** Ramesh Adhikari, Deepika Manduva, Srikrishna V Malayala, Romil Singh, Nitesh K Jain, Keerti Deepika, Thoyaja Koritala

**Affiliations:** 1 Hospital Medicine, Franciscan Health, Lafayette, USA; 2 Geriatrics, Brown University, Providence, USA; 3 Internal Medicine, Temple University Hospital, Philadelphia, USA; 4 Critical Care, Mayo Clinic, Rochester, USA; 5 Critical Care Medicine, Mayo Clinic, Mankato, USA; 6 Pediatrics/Translational Research, Thomas Jefferson University, Philadelphia, USA; 7 Internal Medicine, Mayo Clinic Health System, Mankato, USA

**Keywords:** pneumomediastinum, effects of vaping, e-cigarette or vaping use-associated lung injury (evali), spontaneous pneumomediastinum, e-cigarette smoking, cannabinoids, electronic nicotine delivery systems (ends), electronic nicotine delivery systems, flavors, e-smoking

## Abstract

Vaping is the process of inhaling an aerosol produced by heating a liquid or wax containing substances such as nicotine, cannabinoids (e.g., tetrahydrocannabinol, cannabidiol), flavoring, and additives (e.g., glycerol, propylene glycol). The presence of air or gas in the mediastinum is pneumomediastinum.

We present a rare case of vaping-induced spontaneous pneumomediastinum. A young 20-year-old female patient with a history of vaping and no past medical history presented with acute chest pain to an emergency room. The urine drug screen was positive for cannabinoids. Imaging studies - chest x-ray and computed tomography of the chest - showed pneumomediastinum. The patient was discharged after a day of observation and counseling to quit vaping.

## Introduction

Electronic cigarette (e-cigarette) or vaping products were introduced into United States of America (USA) markets in 2007; since then, sales rose rapidly [1]. Vaping is the process of inhaling an aerosol that is produced by heating a liquid or wax containing substances such as nicotine, cannabinoids (e.g., tetrahydrocannabinol [THC], cannabidiol), flavoring, and additives (e.g., glycerol, propylene glycol) using an e-cigarette [2]. Few regulations exist to control the quality and composition of the ingredients used in e-cigarettes and e-liquids, including solvents [3].

The first cases of e-cigarette or vaping product use-associated lung injury (EVALI) were reported to the Centers for Disease Control and Prevention (CDC) on August 1, 2019 [4]. EVALI in earlier days was called vaping-related acute lung injury (VpALI) [5]. EVALI is a diagnosis of exclusion with no specific diagnostic test [6].

Vaping is also associated with spontaneous pneumomediastinum (SPM) [7-9], spontaneous pneumothorax [10], pneumorrhachis [11], and diffuse alveolar hemorrhage [12]. It is important to elicit the history of vaping and be vigilant in patients with the above presentations [13].

Pneumomediastinum or mediastinal emphysema is the presence of air or gas in the mediastinum [14]. There are two types of pneumomediastinum-spontaneous and traumatic. SPM can be with or without underlying lung disease (primary or secondary). SPM is commonly seen in thin and tall young males [15]. The incidence of SPM in young adults admitted with unclear chest pain or dyspnea in one screening study was 1:368 [16].

We present a case of SPM secondary to vaping in a 20-year-old female patient.

## Case presentation

A 20-year-old caucasian female with a one-year history of vaping, history of ovarian cyst, and umbilical hernia repair presented in the Emergency room for chest pain evaluation from the previous night. She went to bed that night, and when she woke up, the pain in the chest was still there. She described the chest pain as stabbing and pressure-like. She denied radiation of the pain to the back, neck, or left arm. Chest pain worsened with activity and was associated with shortness of breath with deep breathing. The patient denied any relieving factors.

The patient denied any nausea, vomiting, bowel or bladder symptoms, recent travel, fever, chills, or cough.

The patient admitted to smoking e-cigarettes for the last year and used different flavors. She denied smoking cigarettes or using recreational drugs. She denied using marijuana with vaping products.

Laboratory: Complete metabolic panel; complete blood picture was normal. Alpha antitrypsin levels are normal. COVID-19 result RT-PCR is negative. Urine drug screen is positive for opiates and cannabinoids. The urine hCG test is negative. An electrocardiogram showed sinus rhythm with premature ventricular complexes.

Imaging

**Figure 1:**
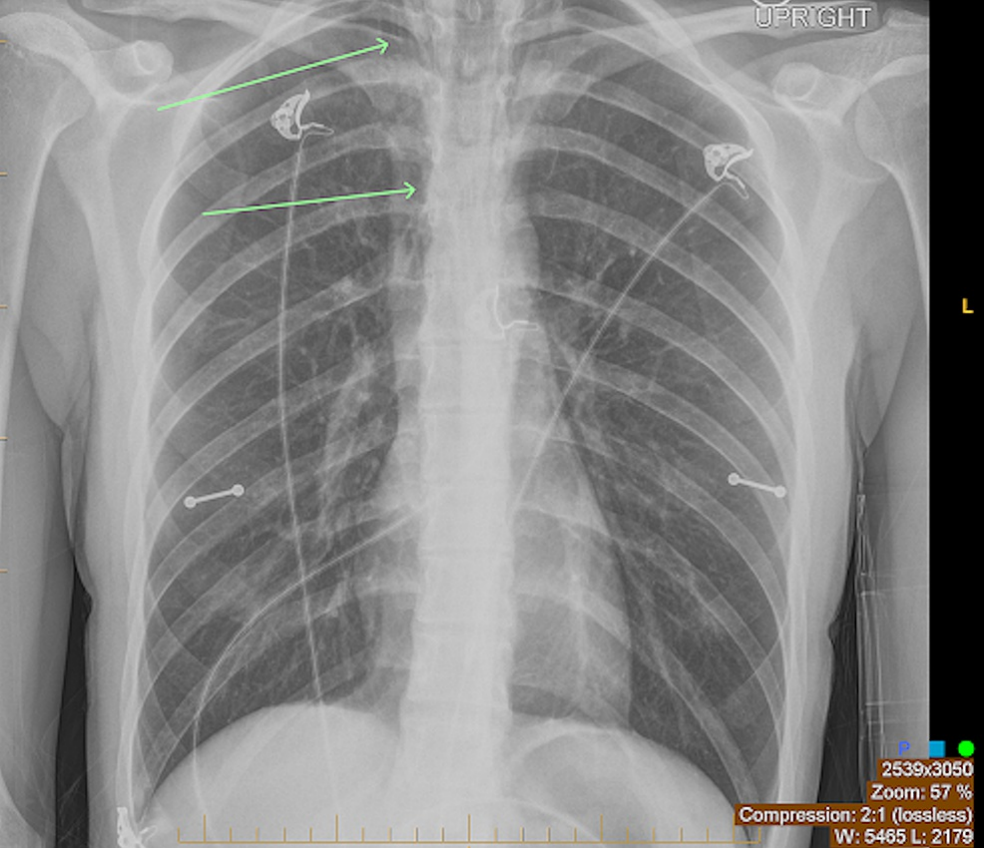
Chest x-ray showing pneumomediastinum.

Chest x-ray showed vertical lucencies in the upper thorax region near the clavicular heads and trachea may be consistent with early pneumomediastinum (Figure 1).

**Figure 2:**
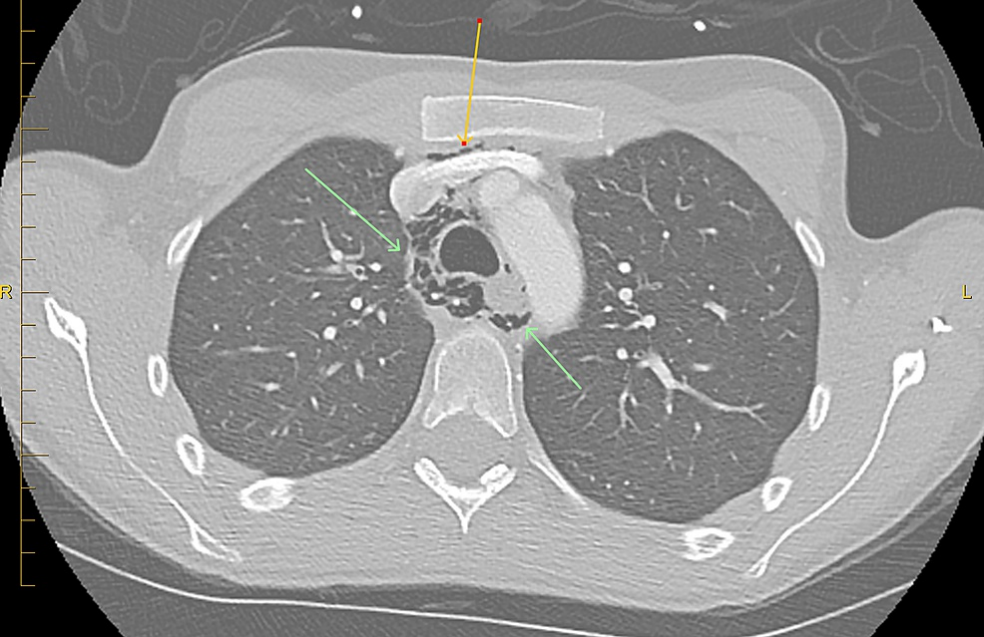
Computerized tomography of chest showing pneumomediastinum (axial view).

**Figure 3:**
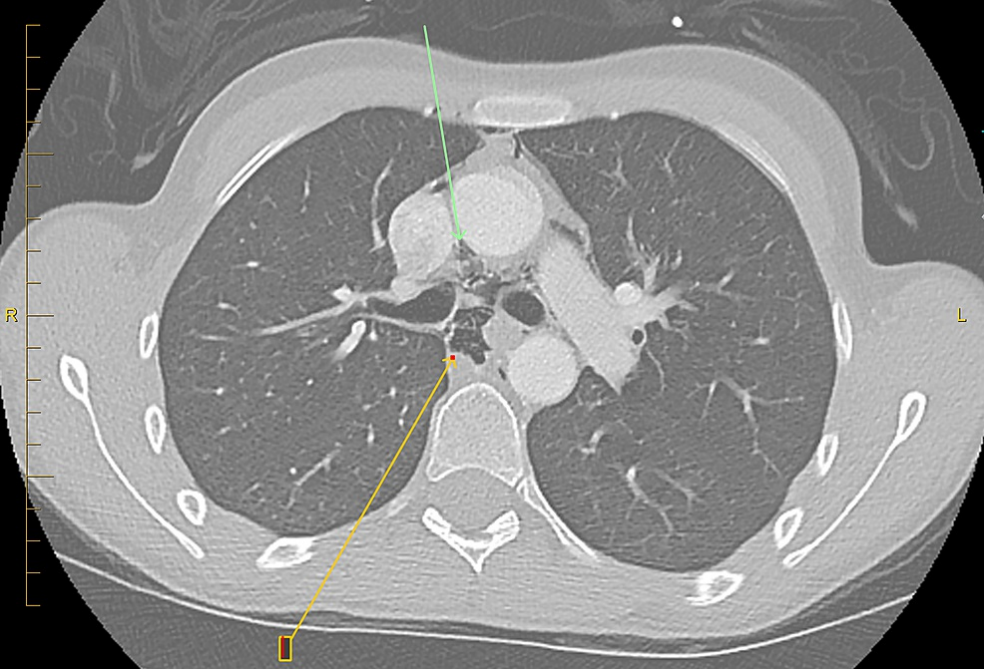
Computerized tomography of chest showing pneumomediastinum.

CT angiogram of the chest with and without contrast to rule out pulmonary embolism showed no pulmonary embolism (Figures 2, 3). Pneumomediastinum is present with air dissecting into the lower neck and about the distal esophagus.

Esophagogram with contrast showed esophageal motility and mucosa are normal without evidence of extravasation, ulcer stricture, or mass. Barium flowed freely into the stomach, and there was no evidence of hiatal hernia or reflux. One minute 20 seconds of fluoroscopy time were provided.

The patient was diagnosed with SPM secondary to vaping nicotine and/or marijuana with a rupture of the pulmonary mediastinal bleb. The next day chest x-ray showed improvement in pneumomediastinum. A detailed history of vaping was asked. The patient admitted to only vaping nicotine and using various flavors - menthol, banana, strawberry, and guava.

A pulmonologist evaluated the patient during the hospitalization and was discharged home with a follow-up chest x-ray in four weeks. The patient was counseled about the risks of vaping, including EVALI.

## Discussion

SPM occurs when small alveoli rupture and air leaks from the alveoli into the bronchovascular sheath [17]. Given the negative pressure in the mediastinum, air flows from the lung parenchyma along the vascular sheaths to the hilum and then into the mediastinum. Air can leak into the pericardium causing pneumopericardium and into the spinal canal causing pneumorrhachis. Individuals inhale aerosolized liquid from e-cigarettes. In order to inhale a large amount of aerosol from the device, they increase their intrathoracic volume. Due to this over inhalation, they tend to forcefully exhale, so as to decompress the lungs. Forceful expiration against the closed glottis increases the intrathoracic pressure, leading to rupture of alveoli and leaking of air along the bronchovascular sheath to the mediastinum.

The main trigger of SPM is acute asthma exacerbation (20%-30%) followed by lower respiratory infections (10%-20%) [18]. Other predisposing conditions included trauma, Valsalva maneuver, vomiting, coughing, esophageal rupture, aspiration of foreign body, choking, barotrauma, dental extraction, inhalation of helium from balloons, use of e-cigarettes, and illicit inhalation drugs.

The most common presenting symptoms of pneumomediastinum are chest pain (55%), shortness of breath (40 %), cough (32%), neck pain (17%), odynophagia (14%), and dysphagia (10%) [19]. Subcutaneous emphysema in the neck and precordial area, Hamman sign suggest pneumomediastinum [20]. Chest x-ray shows bubbles of gas around the mediastinum, clearly seen above the heart on the left side, often extending into the neck or chest wall. Ultrasound of the chest and Computer Tomography (CT) of the chest are other modalities of imaging to identify pneumomediastinum. Pneumothorax and esophageal perforation may occur along with SPM. Tension pneumomediastinum, pneumopericardium, and cardiac tamponade are the possible complications from SPM. 

In patients with uncomplicated SPM, rest, analgesics, and avoidance of triggers are advised. Treatment of the underlying lung disease and oxygen therapy as needed are recommended. SPM usually resolves in 2-15 days without consequences. Recurrence is less than 5% and recurrences are benign [21].

Three similar vaping-induced pneumomediastinum cases have been reported in the literature so far [7-9].

Marasco et al. reported a 17-year-old case of SPM occurring in a healthy young man after his first e-cigarette smoking experience [7]. Burgwardt et al. presented a 25-year-old male with no past medical history presented with chest pain with a diagnosis of SPM. The patient had a history of vaping and at a social gathering, he utilized his e-cigarette in much greater frequency than normal [8]. Alam et al. presented a case of the spontaneous mediastinum in a 22-year-old male patient with a history of vaping and no past medical history. He was positive for Influenza B a week before the presentation [9].

All three case reports presented chest pain with vaping history and SPM diagnosis. Patients are young individuals with no past medical history, which is similar to our case presentation. Our patient is a female patient with vaping history and no past medical history. All patients are asked to quit smoking e-cigarettes and have a follow-up imaging. SPM is usually self-limiting.

It is important to obtain a thorough history of the use of e-cigarettes in a young patient with chest pain presentation and have pneumomediastinum as one of the differential diagnoses.

## Conclusions

Physicians and other health care providers should inquire about e-cigarette use in all patients, especially in young individuals. Awareness about EVALI, an association of Vaping with SPM, spontaneous pneumothorax, pneumorrhachis, and diffuse alveolar hemorrhage among physicians and health care providers helps with identifying the triggers for underlying causes early. A detailed history about vaping products and substrates used for vaping should be included in history taking. Counseling patients about the risks of vaping and encouraging them to quit and providing necessary education will be key in preventing vaping-related medical conditions.
